# Efficacy and safety of early target-controlled plasma volume replacement with a balanced gelatine solution versus a balanced electrolyte solution in patients with severe sepsis/septic shock: study protocol, design, and rationale of a prospective, randomized, controlled, double-blind, multicentric, international clinical trial: GENIUS—Gelatine use in ICU and sepsis

**DOI:** 10.1186/s13063-021-05311-8

**Published:** 2021-06-02

**Authors:** Gernot Marx, Kai Zacharowski, Carole Ichai, Karim Asehnoune, Vladimír Černý, Rolf Dembinski, Ricard Ferrer Roca, Dietmar Fries, Zsolt Molnar, Peter Rosenberger, Manuel Sanchez-Sanchez, Tobias Schürholz, Tamara Dehnhardt, Sonja Schmier, Elke von Kleist, Ute Brauer, Tim-Philipp Simon

**Affiliations:** 1 Klinik für Operative Intensivmedizin und Intermediate Care, Universitätsklinikum RWTH Aachen, Pauwelsstraße 30, 52074 Aachen, Germany; 2 Klinik für Anästhesiologie, Intensivmedizin und Schmerztherapie, Universitätsklinikum Frankfurt/Main, Goethe Universität, Theodor-Stern-Kai 7, 60590 Frankfurt/Main, Germany; 3 Université Côte d’Azur Service de Réanimation Polyvalente, Hôpital Pasteur 2 – CHU de Nice, 30 Voie Romaine, 06000 Nice, France; 4 Université de Nantes, CHU – L’Hôtel Dieu, 1, Place Alexis Ricordeau, 44093 Nantes Cedex 1, France; 5 Krajská zdravotní, a.s., Masarykova nemocnice v Ústí nad Labem, o.z., Sociální péče 3316/12A, 401 13 Ústí nad Labem, Czech Republic; 6 Klinik für Intensivmedizin und Notfallmedizin, Klinikum Bremen-Mitte, St. Jürgen-Straße 1, 28177 Bremen, Germany; 7 Servicio de Medicina Intensiva, Hospital Universitari Vall d’Hebron, Passeig Vall d’Hebron, 119-129, 08035 Barcelona, Spain; 8 Allgemeine und Chirurgische Intensivstation, Medizinische Universität Innsbruck, Anichstraße 35, 6020 Innsbruck, Austria; 9 School of Medicine, Institute for Translational Medicine, University of Pécs, 12 Szigeti St, Pécs, 7624 Hungary; 10 Faculty of Medicine, Department of Anaesthesiology and Intensive Therapy, Poznan University for Medical Sciences, 49 Przybyszewskiego St, 60-355 Poznan, Poland; 11 Universitätsklinik für Anästhesiologie und Intensivmedizin, Universitätsklinikum Tübingen, Hoppe-Seyler-Straße 3, 72076 Tübingen, Germany; 12 Hospital Universitario La Paz, Cantoblanco-Carlos III/Hospital La Paz Institute for Health Research (IdiPAZ), Paseo de la Castellana, 261, 28046 Madrid, Spain; 13 Klinik und Poliklinik für Anästhesiologie und Intensivtherapie, Universitätsmedizin Rostock, Schillingallee 35, 18057 Rostock, Germany; 14 Medical Scientific Affairs, B. Braun Melsungen AG, Carl-Braun-Straße 1, 34212 Melsungen, Germany

**Keywords:** Colloids, Gelatine, Critically ill, Sepsis, Resuscitation, Fluid management, Capillary leakage

## Abstract

**Background:**

Sepsis is associated with capillary leakage and vasodilatation and leads to hypotension and tissue hypoperfusion. Early plasma volume replacement is required to achieve haemodynamic stability (HDS) and maintain adequate tissue oxygenation. The right choice of fluids to be used for plasma volume replacement (colloid or crystalloid solutions) is still a matter of debate, and large trials investigating the use of colloid solutions containing gelatine are missing. This study aims to investigate the efficacy and safety of plasma volume replacement using either a combined gelatine-crystalloid regime (1:1 ratio) or a pure crystalloid regime.

**Methods:**

This is a prospective, controlled, randomized, double-blind, international, multicentric phase IV study with two parallel groups that is planned to be conducted at European intensive care units (ICUs) in a population of patients with hypovolaemia in severe sepsis/septic shock. A total of 608 eligible patients will be randomly assigned to receive either a gelatine-crystalloid regime (Gelaspan® 4% and Sterofundin® ISO, B. Braun Melsungen AG, in a 1:1 ratio) or a pure crystalloid regime (Sterofundin® ISO) for plasma volume replacement. The primary outcome is defined as the time needed to achieve HDS. Plasma volume replacement will be target-controlled, i.e. fluids will only be administered to volume-responsive patients. Volume responsiveness will be assessed through passive leg raising or fluid challenges. The safety and efficacy of both regimens will be assessed daily for 28 days or until ICU discharge (whichever occurs first) as the secondary outcomes of this study. Follow-up visits/calls will be scheduled on day 28 and day 90.

**Discussion:**

This study aims to generate evidence regarding which regimen—a gelatine-crystalloid regimen or a pure crystalloid regimen—is more effective in achieving HDS in critically ill patients with hypovolaemia. Study participants in both groups will benefit from the increased safety of target-controlled plasma volume replacement, which prevents fluid administration to already haemodynamically stable patients and reduces the risk of harmful fluid overload.

**Trial registration:**

The European clinical trial database EudraCT 2015-000057-20 and the ClinicalTrials.gov Protocol Registration and Results System ClinicalTrials.gov NCT02715466. Registered on 17 March 2016.

**Supplementary Information:**

The online version contains supplementary material available at 10.1186/s13063-021-05311-8.

## Background

Sepsis is one of the major global health issues and is considered a leading cause of death in noncoronary intensive care units (ICUs) [1–3]. Sepsis is associated with increased microvascular permeability (capillary leakage) and vasodilatation that leads to interstitial oedema and intravascular fluid deficit [4]. As a result, tissue perfusion becomes inadequate, and the oxygen supply is decreased, which eventually results in multi-organ failure (MOF) and death [5]. To compensate for the intravascular fluid deficit, suitable fluids may be given intravenously (in the following referred to as plasma volume replacement) to establish a stable blood pressure and a consistent cardiac output with consecutive organ perfusion, i.e. haemodynamic stability (HDS). Early initial plasma volume replacement is recommended in septic patients to maintain appropriate cardiac output and tissue oxygenation [6, 7]. Plasma volume replacement should be target-controlled and individualized to minimize the risk of harmful fluid overload associated with worse outcomes [8–10]. Therefore, as recent data suggest, plasma volume replacement should be guided by flow-based parameters or passive leg raising (PLR), which are useful bedside tests for volume depletion and/or volume responsiveness [11]. The PLR manoeuvre functions as a reversible self-volume challenge of approximately 250 mL of blood. Haemodynamic changes occurring within 30 to 90 s after PLR reliably predict volume responsiveness in a variety of clinical settings [12, 13].

Fluids suitable for plasma volume replacement are either crystalloid solutions (composed of water and electrolytes) or colloid solutions (containing macromolecules dissolved in an electrolyte solution). Crystalloid solutions diffuse easily into the interstitial space (IS), especially in the case of capillary leakage, and may potentially cause tissue oedema [14]. In contrast, colloid solutions contain macromolecules that are unable to pass semipermeable biological membranes and exert colloid-osmotic pressure, retaining water into the intravascular space (IVS). Several studies have shown that colloids remain in the IVS regardless of their molecular weight or the degree of capillary leakage [15–17]. Thus, it is postulated that the amount of fluid needed for plasma volume replacement is lower with colloid solutions than with crystalloid solutions, and consecutive studies may speculate that less fluid is needed to achieve HDS using this method [14, 18].

Currently available colloid solutions for plasma volume replacement contain either human albumin, hydroxyethyl starch (HES, made from potato or maize starch) or gelatine (produced by hydrolyses of bovine collagen). Human albumin is rare and expensive because it is obtained from human sources. HES-containing solutions are contraindicated in septic patients because a variety of clinical trials have suggested negative outcomes with respect to renal function and mortality after the use of HES in critically ill patients [18–20]. Gelatine is thus the only clinically relevant colloid for the treatment of hypovolaemia in septic patients. Since very few trials have been conducted with gelatine in this patient population, the benefit or harm of gelatine cannot be determined from the current evidence [21–24]. Uncertainty about appropriate plasma volume replacement in septic patients, therefore, persists [25]. The current guidelines recommend crystalloids for initial plasma volume replacement in critically ill patients [7]. If colloids are used for initial plasma volume replacement in critically ill patients, they are given in a 1:1 to 1:2 ratio with crystalloids in routine clinical practice.

### Study objective

This study aims to investigate the efficacy of early target-controlled plasma volume replacement using a combined gelatine-crystalloid regime in comparison to a pure crystalloid regime in achieving haemodynamic stability (HDS) in patients with severe sepsis/septic shock (the protocol of this study refers to the former definitions of sepsis, severe sepsis and septic shock as implemented in routine clinical practice at the time of enrolment start) and will provide data on the safety and efficacy of the applied fluid regimens.

## Methods/design

### Study design

This is a prospective, controlled, randomized, double-blind, international, multicentric phase IV study with two parallel groups aiming to investigate the efficacy and safety of early target-controlled plasma volume replacement using either a combined gelatine-crystalloid regime (1:1 ratio) or a pure crystalloid regime to achieve haemodynamic stability (HDS) in patients with severe sepsis/septic shock. A total of 608 eligible patients will be randomly assigned to receive the gelatine-crystalloid regimen or the pure crystalloid regime. The time needed to achieve HDS will be recorded as the primary outcome, and the patients will be examined daily during the subsequent 28 days or until ICU discharge, whichever occurs first, to assess safety and efficacy parameters. Follow-up visits/calls will be scheduled on day 28 and day 90 after randomization. To avoid fluid overload, the administration of investigational medicinal products (IMPs) will be target-controlled. Volume responsiveness will be assessed via the passive leg raising (PLR) manoeuvre or via fluid challenges of up to 500 mL of IMP.

Severe sepsis/septic shock will be diagnosed according to the American College of Chest Physicians/Society of Critical Care Medicine (ACCP/SCCM) criteria and definitions [1, 26]. These criteria and definitions were generally accepted to diagnose severe sepsis/septic shock at the time of protocol development. After the approval of the study protocol, a new definition of sepsis was published [27]; however, considering the current clinical routine, it was decided to proceed with the study with the approved protocol based on ACCP/SCCM criteria and definitions. Concordance of diagnoses using ACCP/SCCM criteria and the new definitions of sepsis will be checked during analysis.

The populated SPIRIT checklist for this study is provided as Additional file 1.

### Study population, eligibility criteria

The study will be conducted at European ICUs in a population of male and female patients aged ≥ 18 years with hypovolaemia in severe sepsis/septic shock diagnosed at ICU admission or during the ICU stay. Participating sites are accessible at ClinicalTrials.gov.

Hypovolaemia will be indicated by volume responsiveness, i.e. mean arterial pressure (MAP) or stroke volume index (SVI) increase of > 10% after PLR or fluid challenge (see the “Enrolment” section). Patients must be enrolled within 90 min after diagnosis of severe sepsis or septic shock at the ICU or during their ICU stay. The following additional inclusion criteria apply: body weight ≤ 140 kg, antibiotic therapy already started prior to randomization, negative pregnancy test, and signed informed consent/deferred consent.

Reasons for exclusion are the administration of HES, dextran solutions, or > 500 mL of gelatine solutions within 24 h prior to randomization; death expected within the next 48 h (moribund patients as defined by American Society of Anesthesiologists (ASA) ≥ class V [28]); expected need for pressure infusions; confirmed acute SARS-CoV-2 (COVID-19) infection, a requirement for renal support; renal failure; severe congestive cardiac dysfunction; therapeutic heparin medication due to chronic coagulation disease/anticoagulation medication (i.e. partial thromboplastin time > 60 sec); acute burn injuries; severe general oedema; hypersensitivity to the active substance or ingredients of the IMPs; hypersensitivity to galactose-alpha-1,3-galactose (alpha-Gal) or known allergy to red meat (mammalian meat) and offal; hypervolaemia/hyperhydration; hyperkalaemia; hypercalcaemia; metabolic alkalosis; or simultaneous participation in another interventional clinical trial.

### Investigational medicinal products (IMPs), open-label medication

The investigational medicinal test product Gelaspan® 4% (B. Braun Melsungen AG) is a clear, colourless or slightly yellowish 4% succinylated gelatine solution in an isotonic, fully balanced electrolyte solution. Sterofundin® ISO (B. Braun Melsungen AG), which is a colourless aqueous fully balanced electrolyte solution, serves as an investigational medicinal reference product and for both treatment groups as a noninvestigational medicinal product (open-label medication).

Both products are solutions for infusion provided in 500-ml ready-to-use plastic bottles made of polyethylene (Ecoflac plus®). Patients will receive blinded IMP (Gelaspan® 4% or Sterofundin® ISO) and open-label medication (Sterofundin® ISO) in a 1:1 ratio (i.e. one bottle of blinded IMP, followed by one bottle of open-label medication, and so on). Thus, patients will either receive a combined gelatine-crystalloid regimen (Gelaspan® 4% and Sterofundin® ISO) or a pure crystalloid regime (Sterofundin® ISO only). Since the composition of Sterofundin® ISO reflects the current state of research and current recommendation for fluid replacement in septic patients [29, 30], it is considered a suitable reference for this study.

### Randomization, blinding, and unblinding

Eligible patients will be randomized to either treatment in a 1:1 ratio, stratified for study site and RBC pretreatment (within 24 h prior randomization). Randomization will be based on the patient number, which is assigned at enrolment and indicates country, study site and red blood cell (RBC) pretreatment. A list assigning treatment to each patient number (randomization list) will be generated prior to the initiation of the study using random permuted blocks by an independent biometrician, and sets of emergency envelopes will be prepared.

IMP (Gelaspan® 4% or Sterofundin® ISO) will be blinded, but the noninvestigational medicinal product (Sterofundin® ISO) will be provided in open-label bottles. The blinding of IMP will be performed in advance by the sponsor as a part of the sample manufacturing process. Due to the yellowish colour of gelatine solutions, blinding cannot be assured by covering and labelling alone but additionally requires administration of the study medication via orange infusion lines.

Blinded IMP (Gelaspan® 4% or Sterofundin® ISO) and open-label medication (Sterofundin® ISO) will be supplied by the sponsor in one single box per patient, containing 16 bottles of blinded IMP and 16 bottles of open-label medication (covering the possible maximal amount of IMP and open-label medication required by each patient). To ensure the correct administration sequence, the blinded bottles and open-label bottles will be arranged alternately in the box, starting with the blinded IMP. The bottles must be administered successively. To allow for correct treatment assignments, boxes are labelled with the patient number according to the randomization list before shipment to the study site.

Except for emergency reasons and, if necessary, for review of the unblinded data by the Data Safety Monitoring Board (DSMB, see the “Safety evaluation and reporting of adverse events” section), the study will only be unblinded after the closure of the database and determination of the analysis populations in a blind data review meeting.

### Interventions and procedures

Study phases, interventions, and assessments are summarized in the GENIUS study flow diagram (Table 1).

**Table 1:** GENIUS study flow diagram

Timepoint	Study period				Follow-up period	
	Enrolment	Randomization	Post-randomization		Follow-up	
	− 90 min	*t* = 0	Treatment phase, *t* = 0 to max. 48 h	Daily assessments, 48 h until ICU discharge or day 28	Day 28	Day 90
**Enrolment:**						
Eligibility	x					
Informed consent	x					
Initial PLR manoeuvre	x					
Randomization		x				
**Interventions:**						
IMP administration			x			
**Assessments:**						
**Demographics and anamnesis**^a^						
Demographic data		x				
Anamnesis		x				
Morbidity scores and temperature		x	x^b^	x^b^		
Sepsis		x	x	x		
**Primary outcome parameter**						
Time to HDS			x			
**Safety parameters**^a^						
Renal function		x^c^	x	x		
Coagulation		x	x	x		
Hepatic function		x	x	x		
Adverse Events			x	x		
Need for blood products			x	x		
Concomitant therapies/medication			x	x		
**Efficacy parameters**^a^						
IMP and open-label medication			x			
Crystalloids for further volume treatment			x	x		
Fluid balance			x	x		
Volume responsiveness			x			
Haemodynamic parameters			x	x		
Tissue oxygenation and acid-base balance		x^d^	x	x		
**Clinical outcome parameters**^a^						
Fulfilment of ICU discharge criteria			x	x		
ICU/hospital LOS			Cumulative			
Indication for RRT			Whenever applicable			
Days on RRT			Cumulative			
Infection/antibiotic-free days			Cumulative			
Vasopressor-free days			Cumulative			
Ventilator-free days			Cumulative			
Study termination			Whenever applicable			
**Follow-up parameters**^a^						
Colloid therapy^e^					x	
Last available serum creatinine (SCr)					x	
Mortality, cause of death					x	x
Quality of life (QoL)^f^						x
New RRT, kidney disease						x

^a^A listing specifying the parameters determined is provided in section Methods/design, subsection Outcome measures

^b^Except Apache II

^c^Except urine output, need and indication for RRT

^d^Lactate only

^e^Date and drug applied, retrospectively from ICU discharge until Day 28 or hospital discharge, whichever occurs first

^f^Taking into account the patient population, the patients’ condition and the effort required to assess QoL, it was decided to collect data on QoL on Day 90 only

#### Enrolment

Informed consent must be obtained from all patients or their legal representatives, authorized persons or relatives, depending on local regulations. Since the patients will not be able to consent personally and the time required until enrolment in the study will be too short to receive informed consent from a legal representative, authorized person or a family member, informed consent will be obtained within 90 min, according to the deferred consent procedure approved by the respective ethics committees.

Inclusion and exclusion criteria will be checked, and the patient will undergo the PLR manoeuvre or fluid challenge (MAP or SVI increase of > 10%) for an initial test of volume responsiveness.

As soon as possible, the patients/family members/legal representatives will be advised that they have the right to withdraw from the study at any time without prejudice and may be withdrawn at the investigator's/sponsor’s discretion at any time when it is considered to be in the interest of the patient. Personal consent will be obtained from each patient after regaining competence in decision making or by a family member or legal representative in cases where recovery is not achieved during the study’s duration according to local legal requirements.

#### Treatment phase

Patients will be randomized with the start of alternate intravenous infusions of blinded IMP (gelatine solution or balanced electrolyte solution) and open-label medication (balanced electrolyte solution). Study medication will be administered until the achievement of first/initial HDS, administration of maximum daily dose (30 ml/kg for IMP and open-label medication, each) or 48 h after randomization, whichever occurs first. During treatment with fluids, MAP will be continuously titrated to a value greater than 65 mmHg with norepinephrine. HDS will be defined as MAP ≥ 65 mmHg and fulfilment of at least two of the following criteria:

- Decrease in arterial lactate within the last 6 h > 10% or lactate < 2.4 mmol/L
- Urine production > 0.5 mL/kg/h
- Central venous oxygen saturation (ScvO_2_) > 70%

Volume responsiveness will be assessed via changes in MAP (MAP increase > 10% compared to baseline) after PLR, performed 30 min after fluid administration at the latest (maximum 2 bottles of study medication, i.e. 1 bottle of IMP and 1 bottle of open-label medication), as long as no haemodynamic monitoring system is in place. As soon as the system is in place (not later than 6 h after randomization), volume responsiveness will be assessed via changes in SVI (SVI increase > 10%) determined directly after administration of each bottle of study medication (i.e. upon a fluid challenge of 500 mL) or upon PLR.

If the patient is no longer volume responsive, administration of study fluids will be stopped, and criteria for HDS will be checked. In case HDS is not established, inotropic therapy (preferably dobutamine) will be given. The administration of study medication will be continued if the patient is volume responsive again (tested via PLR; a fluid challenge can be used only in exceptional cases where the patient’s condition precludes PLR). If the criteria for HDS are fulfilled, treatment with the study medication will be temporarily stopped, and the patient will be further monitored for 4 h by means of blood gas analysis and examination of urine output. If during these 4 h the patient remains haemodynamically stable (criteria fulfilment), there is no need to increase inotrope and/or vasopressor therapy due to sepsis, and a maximum of 1 L of additional study fluids is administered, then the patient will be considered stable, and treatment with study fluids will be stopped. Otherwise, treatment with the study medication will be resumed after testing volume responsiveness and inotropic therapy as required. Fluid needs beyond the total maximum daily dose of study medication, after the initial achievement of HDS or after 48 h of randomization (whichever occurs first), will solely be adjusted by applying a crystalloid solution selected by the treating physician until ICU discharge or day 28 (whichever occurs first). A schematic overview of the treatment phase is provided in the Additional file 2.

#### Daily assessments

Patients will be examined daily starting 48 h after randomization until ICU discharge or day 28 (whichever occurs first), and safety and efficacy variables will be recorded (see the “Methods/design” section, specifically, the “Outcome measures” section).

#### Follow-up (FU)

FUs will be conducted 28 days and 90 days after randomization using a follow-up letter/e-mail or call. If contact is not possible, hospital records will be checked for follow-up information. On day 28, information on mortality, colloid use, and the last serum creatinine value will be assessed. On day 90, mortality, quality of life (measured by the health-related quality of life questionnaire, EQ-5D-5L™, EuroQol Group, [31]) and new kidney disease status will be recorded. If a new renal replacement therapy (RRT) or kidney disease occurred by day 90, any concerned patients might be personally visited and interviewed by the investigator. This optional visit intends to further assess the potential relatedness of kidney injury and study treatment and is explicitly mentioned in the patient information provided to each study participant.

### Study and treatment duration

#### Duration per patient

The study starts with randomization (i.e. the start of IMP treatment) and ends with ICU discharge or day 28, whichever occurs first. The treatment period will not exceed 48 h.

The patients will be followed up on days 28 and 90 after randomization.

#### Duration of the whole study

The study started in the first quarter of 2016. A recruitment time of 5.5 years is expected.

### Outcome measures

#### Primary outcome

The primary objective of this study is to investigate the efficacy of early target-controlled plasma volume replacement using a gelatine-crystalloid regime compared to a pure crystalloid regime in achieving HDS in severe sepsis/septic shock patients with hypovolaemia. This will be assessed by measuring the time elapsed between the start of IMP administration and first/initial HDS as the primary outcome.

#### Secondary outcomes

The secondary objective of this study is to investigate the safety and efficacy of the applied fluid regimes. Safety, efficacy, clinical outcome, and follow-up parameters will be determined as secondary outcomes (see Table 1, GENIUS Study Flow Diagram). A list of all secondary outcomes is provided as Additional file 3.

### Concomitant medication, therapies

The following concomitant medications are allowed:

- Norepinephrine as vasoactive treatment (titrated to a MAP > 65 mmHg)
- Inotropic treatment (preferably dobutamine)
- Albumin supplementation (not volume replacement) 48 h after randomization until ICU discharge or day 28 (whichever occurs first)
- Crystalloids to cover fluid needs exceeding the total maximum daily dose of IMP and open-label medication during the treatment period (from randomization until the achievement of HDS or 48 h after randomization)
- Blood products (RBCs, fresh frozen plasma (FFP) or platelet concentrates)
- RRT as continuous RRT during the first 48 h after randomization. Thereafter intermitted RRT is allowed.
- Medication as clinically required.

For the whole study duration (i.e. until ICU discharge or day 28, whichever occurs first), the administration of colloids for volume replacement aside from study medication is not allowed.

In the case of protocol deviations, concerned patients will remain in the study for safety reasons but might be excluded from the per protocol set during the blind data review meeting.

### Safety evaluation and reporting of adverse events

Throughout the clinical trial, particular attention will be given to (serious) adverse events ((S)AEs). The investigator must record all AEs in detail, whether serious or not. A Data Safety Monitoring Board (DSMB) that is not involved in the study and consists of two clinicians and a biometrician will review the data generated throughout the study, preferably in a blinded manner. The DSMB may request unblinding for data review. All SAEs have to be reported to the sponsor within 24 h after awareness, and a notification will be sent to the DSMB. All patients enrolled in this clinical trial must be regarded as critically ill patients in a life-threatening state of disease requiring intensive care events associated with the course of organ dysfunctions as a consequence of severe sepsis/septic shock. All clinically significant abnormal laboratory values as a consequence of underlying disease and/or ICU treatment are not subject to expedited reporting. Expedited reporting applies for death, the suspicion of a causal relationship to the IMP applied, clinically significant abnormal laboratory values, or complications that cannot be explained by the underlying disease or ICU treatment.

SAEs with suspicion of causal relationship to the study treatment that are unexpected according to the available summary of product characteristics (SmPC) of Gelaspan® 4% and Sterofundin® ISO have to be considered suspected unexpected serious adverse reactions (SUSAR). SUSARs are subject to expedited reporting. The sponsor will notify the competent authorities, ethics committees (ECs), and all investigators concerned of SUSARs, in line with pertinent legal requirements.

The DSMB will assess the progress, safety data, and the critical efficacy variables of this study if needed. Based on its review, the DSMB will provide the sponsor with recommendations regarding study modification, continuation, or termination. The entire clinical study might be discontinued upon unexpectedly high-frequency SAEs, the occurrence of SUSARs, or an insufficient number of recruited patients.

Individual patients will be withdrawn by the investigator if haemodynamic monitoring cannot be established or if (S)AE (including pregnancy) and clinically significant abnormal laboratory values lead to non-acceptance of study continuation.

### Sample size calculation, planned interim analysis

The sample size was based on the assumption of a difference between the gelatine and crystalloid groups. The following hypotheses need to be tested:

- *H*_0_ (null hypothesis): HDS [gelatine] = HDS [crystalloid]
- *H*_A_ (alternative hypothesis, one-sided): HDS [gelatine] ≠ HDS [crystalloid]

The primary variable, i.e. time to first/initial HDS, was used for sample size calculation. The effect size was estimated based on data from a study comparing the use of HES solution using sodium chloride [20]. Sample size calculation resulted in 253 patients per group, assuming an effect size (difference of means/common standard deviation) of 0.25 (− 2.5 h/10 h), an α-error of 5% (two-sided), and a power of 80%. Considering a drop-out rate of 20%, the sample size was determined to be 304 patients per group. Since effect size calculation is only considered a rough estimate, an interim analysis for sample size recalculation is planned upon the inclusion of 400 patients. If the sample size recalculation after 400 patients results in a number exceeding the total number of 608 patients by an extreme amount, then the study will be stopped for futility.

### Statistics

All programming of tables, figures, listings, and statistical analyses will be performed using a statistical software package. Statistics will be performed following the principles outlined by guideline E9 of the International Conference on Harmonisation (ICH) and will be outlined in detail in the statistical analysis plan (SAP) finalized before closing the database.

All primary and secondary variables will first be examined by exploratory data analysis and descriptively evaluated. In this setting, the evaluation of structural homogeneity of the treatment groups will be performed for quality assurance. The primary endpoint (time to HDS) will be evaluated with a nonparametric statistical test (Mann-Whitney *U* test),) taking into consideration small sample sizes and possible deviation from a normal distribution. The stratification variables ‘site’ and ‘RBC pretreatment’ will be included in the primary analysis as covariates (in a nonparametric analysis of covariance).

Secondary target variables will also be evaluated with nonparametric tests according to their scaling. Therefore, in the case of a small random sample size or an unbalanced condition, exact tests will be used. Nonparametric multivariate analysis of variance (MANOVA) for repeated measurement will be performed.

Further regressions will be performed as applicable. Several subgroup analyses are planned according to strata (RBC pretreatment, sites), administration of gelatine 24 h prior to randomization, septic shock/severe sepsis, APACHE II score [32], SOFA score [33], transfusion, the establishment of HDS after one episode/at least two episodes of sepsis/septic shock, and diagnosis of sepsis/septic shock at ICU admission/during ICU stay.

All tests will be two-sided with an α-error of 5%. Tests of all secondary variables will be carried out in the area of exploratory data analysis, if applicable. Therefore, corresponding *p* values are regarded as exploratory, and no adjustments for multiple testing will be made.

All randomized patients will be included in the primary analysis. Missing values will not be imputed.

Outliers may be identified using stem-leaf plots and frequency distributions, scatter plots, and box plots. For normally distributed data, values more than three standard deviations away from the mean will be considered outliers. Transformation of the data to mitigate the influence of outliers may be considered. If outliers remain, additional analyses excluding these values will be performed.

An intent-to-treat (ITT) analysis and full analysis set (FAS) are planned. Additionally, a per protocol (PP) or valid case analysis set (VCAS) will be performed, excluding all patients without stopping treatment due to adverse reactions and/or severe protocol violations.

### Data registration, monitoring

All data obtained in the context of the clinical trial are subject to data protection. Storage and processing of personal data will be under the provisions set forth by the European Union General Data Protection Regulation (GDPR) 2016/679 and national law. Data processing occurs on the legal basis of the patient’s informed consent to participate in this clinical study or the consent of his/her legal representative/authorized person or relative.

Every effort will be made to collect all data points in the study. The amount of missing data will be minimized by appropriate management of the trial, proper screening of patients, and training of participating investigators and other authorized staff (e.g. nurses), clinical research associates (CRAs), and study managers.

The data generated in this study will be recorded using a computerized system following applicable regulations. The system will generate an individual electronic case report form (eCRF) for each patient participating in the trial. The principal investigator of each study site must ensure the accuracy and completeness of the site data entered in the system using an electronic signature. The eCRF system will guarantee compliance with 21 CFR part 11 [34], data safety, communication security, limited access, and full audit trail.

Authorized, qualified CRAs will visit investigational sites in regular intervals as defined in the monitoring plan to verify adherence to protocol and local legal requirements, perform source data verification, and assist the investigator in his/her study-related activities. An independent audit at the study site may take place at any time during or after the study.

### Ethical and legal considerations

This clinical study will be conducted in accordance with the Declaration of Helsinki. It will be conducted in compliance with the protocol, good clinical practice (GCP) (2001/20/EEC, CPMP/ICH/135/95), designated SOPs, and local laws and regulations relevant to the use of investigational new drugs in the country of conduct. This protocol, in its current version 6.0, has been approved by all competent authorities and ethics committees involved.

During the study, all documents that are subject to review will be provided to the institutional ethics committees by the sponsor or the investigator in line with national provisions. Protocol amendments will be submitted to the concerned ethics committees and competent authorities in line with pertinent regulatory requirements. The list of all involved ethics committees and competent authorities is provided as Additional file 4.

### Responsibilities

Responsibilities of investigators, CRAs, and sponsors of the clinical trial regarding handling and storage of data, planning, assessment, and quality assurance are regulated by the recommendations on ‘ICH Topic E6 Guideline for Good Clinical Practice’ of the International Conference on Harmonisation (ICH) and apply to this clinical trial.

The costs necessary to perform the study have been agreed upon with each investigator and are documented in separate financial agreements that have been signed by the hospital administration, investigator and sponsor prior to the study commencing.

The sponsor, B. Braun Melsungen AG, has taken out subject insurance for all patients taking part in the trial.

### Publication policy

The sponsor and coordinating/principal investigators shall agree on the final study report. It is intended that the results of the study may be published as scientific literature. Following generally recognized principles of scientific collaboration, co-authorship with any sponsor personnel will be discussed before submission of a manuscript to a publisher. The results may also be used in submissions to regulatory authorities. Information developed in this clinical study may be disclosed as required to investigators involved in this study or any appropriate international regulatory authorities (e.g. to comply with reporting obligations of SUSARs). The sponsor will be provided with complete test results, and all data will be developed during this study in pseudonymized form. All patients will be informed about the storage, processing and transfer of pseudonymized personal data generated in this study, and informed consent will be mandatory for participation in this clinical study.

## Discussion

This study aims to provide data contributing to answering the question about the right choice of fluid for plasma volume replacement in patients with severe sepsis or septic shock. In contrast to pragmatic studies investigating the safety and efficacy of HES in critically ill patients, the study design of this clinical trial ensures that gelatine containing investigational test products will be administered in line with the recommendations of the manufacturer as outlined in the product information (e.g. contraindications will be respected). Patients will be included within 90 min after the diagnosis of severe sepsis or septic shock to prevent fluid administration prior to randomization, which might bias the study results. Further, fluid administration will be target-controlled; i.e. fluids will be administered to volume-responsive patients only to minimize the risk of fluid overload. This approach also prevents the administration of colloids to already haemodynamically stable patients, which is contraindicated and a limitation of most trials that investigated plasma volume replacement in critically ill patients so far [35]. Study participants in both groups will benefit from target-controlled plasma volume replacement because it increases safety compared to fluid therapy in normal clinical practice, which is most often guided by inadequate haemodynamic parameters such as central venous pressure [29, 36]. The design of this study adequately considers the safety checklist recently published by Meybohm et al. regarding the planning of prospective randomized clinical trials in the field of acute plasma volume replacement in critically ill patients [37].

Patients with severe sepsis or septic shock are the elected study population since they typically require large amounts of fluids [9] and may benefit from the volume expanding properties of colloids. During the last two decades, sepsis has been described as a form of systemic inflammatory response syndrome (SIRS) caused by a known or suspected microbial invasion of normally sterile parts of the body [38]. Sepsis associated with infection-induced organ dysfunction or tissue hypoperfusion was rated as severe sepsis, and severe sepsis accompanied by hypotension or the need for vasopressors despite adequate plasma volume replacement was defined as septic shock [39]. In 2016, new definitions and clinical criteria for sepsis and septic shock were published. These definitions describe sepsis as life-threatening organ dysfunction caused by a dysregulated host response to infection, thereby emphasizing the severity of this systematic illness [27]. According to the new definition, the diagnosis of sepsis already implies organ dysfunction, and the differentiation between sepsis and severe sepsis becomes superfluous. Nevertheless, this protocol refers to the established definitions of sepsis, severe sepsis and septic shock since they were implemented in routine clinical practice at the time enrolment started. To take into account the updated sepsis definition and recommendations regarding diagnostic parameters [27], concordance of diagnoses using established and new diagnostic parameters will be compared in the analysis.

### Trial status

This clinical study is currently in the enrolment phase. Enrolment started on 11 April 2016, and the estimated completion date is the end of 2021. The study protocol uses the current version 6.0, dated 16 July 2020. The change history is given in the “Ethics approval and consent to participate” section.

## Supplementary Information

**Additional file 1.** SPIRIT 2013 Checklist: Recommended items to address in a clinical trial protocol and related documents.

**Additional file 2.** Schematic overview of the treatment phase.

**Additional file 3.** Secondary outcomes.

**Additional file 4.** List of IECs and CAs.

## Data Availability

Not applicable; no datasets are included in this study protocol.

## Abbreviations

ACCP/SCCM: American College of Chest Physicians/Society of Critical Care Medicine
APACHE: Acute Physiology and Chronic Health Evaluation
ASA: American Society of Anesthesiologists
CRA: Clinical research associate
CRO: Contract Research Organisation
DSMB: Data Safety Monitoring Board
EC: Ethics committee
(e)CRF: (electronic) case report form
FAS: Full analysis set
FFP: Fresh frozen plasma
FU: Follow-up
GCP: Good clinical practice
GENIUS: Gelatine Use in ICU and Sepsis
HDS: Haemodynamic stability
HES: Hydroxyethyl starch
HRQoL: Health-related quality of life
i.a.: Inter alia
i.e.: Id est
ICH: International Conference on Harmonisation
ICU(s): Intensive care unit(s)
IMP: Investigational medicinal product
IS: Interstitial space
ITT: Intent-to-treat
IVS: Intravascular space
LOS: Length of stay
MANOVA: Multivariate analysis of variance
MAP: Mean arterial pressure
MOF: Multi-organ failure
PLR: Passive leg raising
PP: Per protocol
RBC: Red blood cell
RRT: Renal replacement therapy
(S)AE: (Serious) adverse event
SAP: Statistical analysis plan
SCr: Serum creatinine
ScvO_2_: Central venous oxygen saturation
SIRS: Systemic inflammatory response syndrome
SmPC: Summary of product characteristics
SOFA: Sequential Organ Failure Assessment
SUSAR: Suspected unexpected serious adverse reaction
SVI: Stroke volume index
VCAS: Valid case analysis set
