# Supplementary material for: Efficacy and safety of early target-controlled plasma volume replacement with a balanced gelatine solution versus a balanced electrolyte solution in patients with severe sepsis/septic shock: study protocol, design, and rationale of a prospective, randomized, controlled, double-blind, multicentric, international clinical trial: GENIUS—Gelatine use in ICU and sepsis
Source: Trials. 2021 Jun 2;22:376. doi: 10.1186/s13063-021-05311-8 (PMC8170449; doi:10.1186/s13063-021-05311-8)
Supplement: Supplementary file 2 — Additional file 2. Schematic overview of the treatment phase. [file 13063_2021_5311_MOESM2_ESM.pdf]

ENROLMENT AND PATIENT TREATMENT

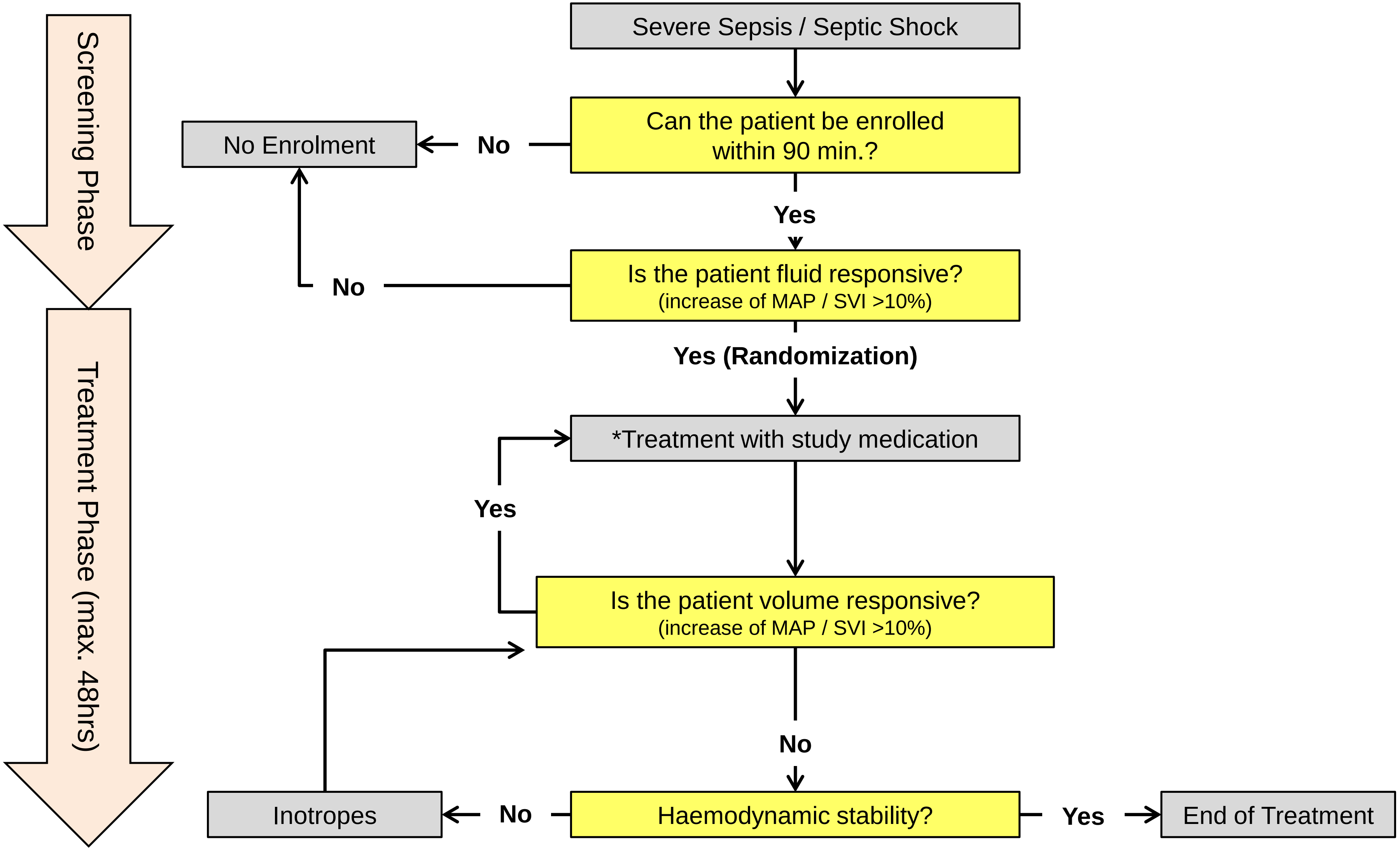

*During the treatment with study fluid MAP is continuously titrated to a value **>65 mmHg** with norepinephrine.
