# Supplementary material for: Efficacy and safety of early target-controlled plasma volume replacement with a balanced gelatine solution versus a balanced electrolyte solution in patients with severe sepsis/septic shock: study protocol, design, and rationale of a prospective, randomized, controlled, double-blind, multicentric, international clinical trial: GENIUS—Gelatine use in ICU and sepsis
Source: Trials. 2021 Jun 2;22:376. doi: 10.1186/s13063-021-05311-8 (PMC8170449; doi:10.1186/s13063-021-05311-8)
Supplement: Supplementary file 3 — Additional file 3. Secondary outcomes. [file 13063_2021_5311_MOESM3_ESM.pdf]

Additional File 2: Secondary outcomes

<u>Safety</u>

Renal function
- Serum creatinine (SCr)
- Serum blood urea nitrogen (BUN)
- Estimated glomerular filtration rate (eGFR) (calculated)
- Need and indication of renal replacement therapy (RRT)
- Urine output
- Kidney Disease Improving Global Outcome (KDIGO) score (calculated) [1]

Coagulation
- Prothrombin time (PT)
- Activated partial thromboplastin time (aPTT)
- International norm ratio (INR)
- Site specific measurements (Austria):
    - Fibrinogen
    - Antithrombin (AT)
    - Platelets absolute

Hepatic function
- Bilirubin

Adverse Events
- (Serious) adverse events ((S)AEs) / reactions ((S)ARs)

Need for blood products
- Number of red blood cell (RBC) units
- Number of fresh frozen plasma units
- Number of other blood products

Concomitant therapies / medication
- Vasopressor therapy
- Inotropic therapy
- Antibiotic therapy
- Nephrotoxic therapy
- Contrast agents
- Anticoagulation therapy

Efficacy

IP and background medication

- Volume needed to achieve first / initial haemodynamic stability (HDS)
- Total volume until 48 h after randomization
- Number of administered bottles

Crystalloids for further volume treatment

- Type of crystalloid and volume

Fluid balance

- Fluid intake
- Fluid output
- Fluid balance

Haemodynamics

- Volume responsiveness (mean arterial pressure (MAP) and stroke volume index (SVI) upon passive leg raising (PLR) or exogenous fluid challenge, respectively)
- Haemodynamic parameters (if available)

Tissue oxygenation and acid base balance

- Arterial (preferably) blood gas analysis (BGA)
  - Partial pressure of carbon dioxide (pCO2)
  - Partial pressure of oxygen (pO2)
  - Bicarbonate
  - Arterial oxygen saturation (SaO2)
  - Haemoglobin
  - Haematocrit
  - Potentia Hydrogenii (pH)
  - Base excess
  - Lactate
  - Serum electrolytes (Sodium, Potassium, Calcium, Chloride)
  - Central venous oxygen saturation (ScvO2)
- Lactate decrease
- Arterial oxygen content (calculated)
- Oxygen delivery (calculated)

Clinical outcome

- Fulfilment of intensive care unit (ICU) discharge criteria
- Length of stay (LOS) in the ICU
- Hospital LOS
- Patient on RTT (Indication for RTT, Days on RRT)

- Patient on invasive mechanical ventilation (Number of ventilator free days)
- Number of infection free days
- Number of antibiotic free days
- Number of vasopressor free days
- Study termination

<u>Follow-up</u>

- Colloid therapy retrospectively from ICU discharge until hospital discharge or day 28, whatever occurs first
- Retrospectively, last available SCr value documented from ICU discharge until day 28 or hospital discharge
- Mortality & cause of death (if applicable)
- Health-related quality of life (HRQoL questionnaire, EQ-5D-5L™, EuroQol Group [2])
- New RRT / kidney disease

<u>Demographics and Anamnesis</u>

Demographic data
- Age
- Gender
- Height
- Weight
- Body surface area (BSA)
- Ethnicity
- Type of patient (e.g. trauma patient, surgical patient)

Anamnesis
- Fluid input in the 24 h prior to randomization
- RBC treatment prior randomization
- Origin of sepsis
- Causative organism of infection
- Medical history

Morbidity scores and temperature
- Acute Physiology And Chronic Health Evaluation (APACHE) II [3]
- Sequential Organ Failure Assessment (SOFA) [4]
- Temperature

Sepsis
- Procalcitonin (optional)
- Severe sepsis / septic shock (2nd, 3rd, … episode)

References:

1. KDIGO 2012 Clinical Practice Guideline for the Evaluation and Management of Chronic Kidney Disease. 2013;3(1), 1-150. https://www.sciencedirect.com/journal/kidney-international-supplements/vol/3/issue/1. Accessed 17 March 2021.
2. Herdman M, Gudex C, Lloyd A, Janssen M, Kind P, Parkin D, et al. Development and preliminary testing of the new five-level version of EQ-5D (EQ-5D-5L). Qual Life Res. 2011;20:1727-36.
3. Knaus WA, Draper EA, Wagner DP, Zimmerman JE. APACHE II: a severity of disease classification system. Crit Care Med. 1985;13(10):818-29.
4. Vincent JL, Moreno R, Takala J, Willatts S, De Mendonça A, Bruining H, et al. The SOFA (Sepsis-related Organ Failure Assessment) score to describe organ dysfunction/failure. On behalf of the Working Group on Sepsis-Related Problems of the European Society of Intensive Care Medicine. Intensive Care Med. 1996;22(7):707-10.
