# Supplementary material for: Efficacy and safety of early target-controlled plasma volume replacement with a balanced gelatine solution versus a balanced electrolyte solution in patients with severe sepsis/septic shock: study protocol, design, and rationale of a prospective, randomized, controlled, double-blind, multicentric, international clinical trial: GENIUS—Gelatine use in ICU and sepsis
Source: Trials. 2021 Jun 2;22:376. doi: 10.1186/s13063-021-05311-8 (PMC8170449; doi:10.1186/s13063-021-05311-8)
Supplement: Supplementary file 4 — Additional file 4. List of IECs and CAs. [file 13063_2021_5311_MOESM4_ESM.pdf]

ADDITIONAL FILE 3 List of IECs and CAs

All institutional Ethic Committees (IECs) (central and local as required per national regulation/law) and Competent Authorities (CAs) involved in the study are listed in the following:

Name, Address, Chair (if applicable) of IECs
Ethik-Kommission an der Medizinischen<br>Fakultät der Rheinisch-Westfälischen<br>Technische Hochschule Aachen (RWTH Aachen), Universitätsklinikum Aachen<br>Pauwelsstraße 30<br>52074 Aachen<br>Germany
Öffentlich-rechtliche Ethikkommission<br>Des Landes Bremen<br>Institut für klinische Pharmakologie<br>Klinikum Bremen-Mitte gGmbH<br>St.-Jürgen-Strasse 1<br>28177 Bremen<br>Germany
Ethikkommission des Fachbereichs Medizin<br>Der Johann Wolfgang Goethe-Universität<br>Theodor-Stern-Kai 7<br>Haus 1, 2. OG, Zi. 207-211<br>60590 Frankfurt an Main<br>Germany
Ethik-Kommission an der Medizinischen Fakultät<br>der Eberhard-Karls-Universität und am Universitätsklinikum Tübingen<br>Gartenstraße 46<br>72074 Tübingen<br>Germany
Ethikkommission an der Medizinischen Fakultät<br>der Universität Rostock<br>St.-Georg-Str. 108<br>18055 Rostock<br>Germany
Ethik-Kommission<br>der Medizinischen Fakultät der<br>Ruhr-Universität Bochum, Sitz Ostwestfalen<br>Georgstr. 11<br>32545 Bad Oeynhausen<br>Germany
Ethik-Kommission<br>der Medizinischen Fakultät der Ruhr-Universität Bochum

Gesundheitscampus 33<br>44801 Bochum<br>Germany
Ethik-Kommission des FB Medizin<br>Medizinisches Lehrzentrum 3. OG<br>Klinikstraße 29<br>35392 Gießen<br>Germany
Universität Duisburg-Essen<br>Ethikkommission<br>Robert-Koch-Str. 9-11<br>45147 Essen<br>Germany
Ethik-Kommission der Ärztekammer Westfalen-Lippe und der Westfälischen<br>Wilhelms-Universität Münster<br>Gartenstraße 210 – 214<br>48147 Münster<br>Germany
CPP SUD MEDITERRANEE V<br>CHU de NICE - Hôpital de CIMIEZ<br>Bâtiment Grand Hôtel - 5ème étage<br>4 avenue Reine Victoria<br>CS. 91179<br>06003 NICE CEDEX 1<br>France
Geschäftstelle der Ethik-Kommission<br>der Medizinischen Universität Innsbruck<br>Innrain 43<br>6020 Innsbruck<br>Austria
Etická komise<br>FN Hradec Králové<br>Sokolská 581<br>500 05 Hradec Králové<br>Czech Republic<br>Chair: Jiří Vortel
Etická komise<br>Masarykova nemocnice v Ústí nad Labem<br>Sociální péče 3316 /12A<br>40113, Ústí nad Labem<br>Czech Republic<br>Chair: Jiří Král
Etická komise<br>Fakultní nemocnice Královské Vinohrady<br>Šrobárova 1150/50<br>100 34 Praha 10<br>Czech Republic

Chair: Prof Jan Pachl	
Etická komise Fakultní Thomayerova nemocnice<br>Videnska 800<br>140 59 Praha 4<br>Czech Republic<br>Chair: Prof. Vladimir Stanek	
Ethics Committee of Clinical Pharmacology of the Medical Research Council, 1051 Budapest, Arany J.u. 6-8,<br>Hungary	
Chair: Víctor Sánchez Margalet<br>CEIm Provincial de Sevilla<br>Hospital Universitario Virgen Macarena<br>Avda. Dr. Fedriani, 3 – 41009 Sevilla<br>Spain	
**Name and Address of CAs**	
Bundesamt für Sicherheit im Gesundheitswesen (BASG)<br>Österreichische Agentur für Gesundheit und Ernährungssicherheit (AGES)	Institut Zulassung & Life Cycle Management/ KPPS<br>Traisengasse 5<br>1200 Wien<br>Austria
Statni ústav pro kontrolu leciv (SUKL)<br>- State Institute for Drug Control (SIDC)<br>Oddělení nežádoucích příhod a klinického hodnocení	Šrobárova 48<br>100 41 Prague 10<br>Czech Republic
Bundesinstitut für Arzneimittel und Medizinprodukte (BfArM)	Referat Klinische Prüfung<br>Kurt-Georg-Kiesinger-Allee 3<br>53175 Bonn<br>Germany
Agence francaise de sanitare des produits de sante (AFSSAPS)	Direction de I`Evaluation des Medicaments et des Produits biologiques (DEMEB)<br>De`partment de I`Evaluation des Medicaments a`Statut particulier et des Essais Cliniques<br>Unite` Essais Cliniques et Loi Huriet<br>143-145 Boulevard Anatole France<br>93200 Saint-Denis<br>France
Országos Gyógyszerészeti és Élelmezés-egészségügyi Intézet (OGYÉI)	Budapest, V., Zrinyi u. 3., Hungary, H-1051<br>Budapest, 5, P.O. Box 450, Hungary, H-1372
Agencia Española de Medicamentos y Productos Sanitarios<br>Área de Ensayos Clínicos	Calle Campezo 1<br>Edificio 8<br>28022 Madrid<br>Spain
